# XIAP upregulates expression of HIF target genes by targeting HIF1α for Lys^63^-linked polyubiquitination

**DOI:** 10.1093/nar/gkx549

**Published:** 2017-06-28

**Authors:** Catherine V. Park, Iglika G. Ivanova, Niall S. Kenneth

**Affiliations:** Institute for Cell and Molecular Biosciences, Faculty of Medical Sciences, Newcastle University, Newcastle upon Tyne NE2 4HH, UK

## Abstract

The cellular response to hypoxia is characterised by a switch in the transcriptional program, mediated predominantly by the hypoxia inducible factor family of transcription factors (HIF). Regulation of HIF1 is primarily controlled by post-translational modification of the HIF1α subunit, which can alter its stability and/or activity. This study identifies an unanticipated role for the X-linked inhibitor of apoptosis (XIAP) protein as a regulator of Lys^63^-linked polyubiquitination of HIF1α. Lys^63^-linked ubiquitination of HIF1α by XIAP is dependent on the activity of E2 ubiquitin conjugating enzyme Ubc13. We find that XIAP and Ubc13 dependent Lys^63^-linked polyubiquitination promotes HIF1α nuclear retention leading to an increase in the expression of HIF1 responsive genes. Inhibition of the Lys^63^-linked polyubiquitination pathway leads to reduced levels of nuclear HIF1α, promoter occupancy, HIF-dependent gene expression and cell viability. Our data reveals an additional and significant level of control of the HIF1 by XIAP, with important implications in understanding the role of HIF1 and XIAP in human disease.

## INTRODUCTION

Hypoxia is the consequence of a failure in oxygen delivery to cells, either as a result of reduced oxygen partial pressures, or disruption to the circulating blood supply (1). Cellular signalling pathways have evolved to enable organisms to adapt to the changing environment and maintain oxygen homeostasis when oxygen levels are limiting (1,2). At the cellular level the response to hypoxia is characterised by a switch in the transcriptional program, resulting in changes in the expression of a large number of genes (1). The main mediator of these changes in gene expression is hypoxia inducible factor (HIF), a family of transcription factors that play key roles in development, physiological processes and pathological conditions (1,2).

The best-characterised form of HIF, HIF1, is a heterodimeric transcription factor composed of HIF1α and HIF1β subunits (2). HIF1 is primarily regulated through the stability of the HIF1α subunit, which is controlled by the action of several proline hydroxylases (PHDs) (3). Following hydroxylation, HIF1α is recognised by the von Hippel-Lindau (VHL) E3 ubiquitin ligase, which promotes the ubiquitination and subsequent degradation of HIF1α by the 26S proteasome (4). The PHDs are inactivated when oxygen levels are reduced, resulting in HIF1α stabilisation and accumulation in the nucleus where it can dimerise with HIF1β to activate target gene expression (5).

Ubiquitination, the covalent attachment of ubiquitin to target proteins, is one of the most versatile post-translational modifications in the cell as it can modify substrate proteins in its monomeric form (monoubiquitination) or be conjugated in the form of ubiquitin chains (polyubiquitination) (6,7). Homogenous, mixed, linear or branched polyubiquitin chains can be generated through successive isopeptide bond formation using any of the seven internal lysines, positioned at amino acid residues 6, 11, 27, 29, 33, 48 and 63, or the initiator methionine (7). Different ubiquitin chain linkages result in distinct polyubiquitin chain topologies creating a range of molecular signals that encode information about the substrate's fate in the cell (7,8).

VHL-dependent regulation of HIF1α is well described, however it is not the only E3 ubiquitin ligase or ubiquitin-mediated mechanism through which HIF1 is regulated. In hypoxic cells ubiquitin chains heavily modify HIF1α, even though the action of VHL is inhibited. Consistent with these observations, unbiased mass spectrometry experiments reveal 25 lysine residues on HIF1α are modified by ubiquitin in cells, but only three of these are required for VHL-dependent degradation (http://thebiogrid.org/109338/protein) (9).

Here we describe a novel, unanticipated role for XIAP, an E3 ubiquitin ligase, in the regulation of HIF1α following hypoxic stress. Depletion of XIAP results in an impaired nuclear accumulation of HIF1α and a resultant decrease in the transactivation of HIF1 target genes. XIAP-dependent regulation of HIF1α is independent of HIF1α stabilisation; instead XIAP directly ubiquitinates HIF1α in a Lys^63^-dependent manner. In addition specific inhibitors of the Lys^63^-ubiquitin conjugation pathway suppress the HIF1-dependent hypoxic response and sensitise cells to hypoxic stress. Our findings suggest that targeting Lys^63^-linked ubiquitination of HIF1α could be used as a therapeutic strategy for diseases with aberrant HIF activity.

## MATERIALS AND METHODS

### Cell lines and culture conditions

U2OS, HEK293 and RCC4 cell lines were grown in Dulbecco's modified eagle medium (Lonza) supplemented with 10% fetal bovine serum (Gibco) and glutamate (Gibco). PC-3 cells were grown in RPMI 1640 medium supplemented with 10% fetal bovine serum and glutamate.

### Treatments

NSC697923 (Sigma), MG132 (Calbiochem), BAY 11-8042 (Calbiochem) and Leptomycin B (Sigma) were prepared in DMSO and added to cells at the concentrations indicated in the figure legends.

### DNA constructs and transfections

pcDNA3-HA-HIF1α (Addgene #18949), pcDNA3-HA-HIF1α P402A P562A (Addgene #18955), pcDNA3-HA-PHD1 (Addgene #18961), pcDNA3-PHD2 (Addgene #19963), pcDNA3-PHD3 (Addgene #18960), pRc/CMV-HA-VHL (Addgene #19999) were gifts from William Kaelin supplied by Addgene. pEBB-His-ubiquitin, pEBB-FLAG-XIAP, pEBB-FLAG-VHL (subcloned from pRc/CMV-HA-VHL into pEBB-Flag), pEBB-N-biotin-HIF-1α, pET15-ubiquilin TUBE (DU 20108 MRCPPU reagents, University of Dundee [mrcppureagents.dundee.ac.uk]), pET28a-6xHis-TAB2 aa 644–692 dimer (DU 23839 MRCPPU reagents), pET28a-6xHis-TAB2 aa 644–692 dimer T674A F675A (DU 46083 MRCPPU reagents). HEK293 transfections were performed using a standard calcium phosphate method. Transfections into U2OS cells were performed using GeneJuice (Invitrogen) according to the manufacturer's instructions.

### siRNA transfection

siRNA duplex oligonucleotides were synthesised by MWG and transfected using Interferin (Polyplus) according to the manufacturer's instructions. siRNA sequences: control—CAG UCG CGU UUG CGA CUG G; XIAP #A—GUG GUA GUC CUG UUU CAG C; XIAP #B—GGA GAU ACC GUG CGG UGC U; HIF1α—GCA UAU AUC UAG AAG GUA U; Ubc13-GGC UAU AUG CCA UGA AUA A.

### Immunoblotting and antibodies

Cell lysates were prepared with modified RIPA lysis buffer (50 mM Tris pH 8.0, 150 mM NaCl, 1% NP40, 0.5% C_24_H_39_NaO_4_, 0.1% SDS) supplemented with protease inhibitors and incubated on ice for 15 min. Lysates were then sonicated in a Biodisrupter Ultra-Sonic Waterbath (Diagenode) (2 × 30 s cycles on high) to shear the genomic DNA and clarified by centrifugation at 13 000 rpm at 4°C for 10 min.

Nuclear and cytoplasmic extracts were prepared from a single sub-confluent 10 cm dish of U2OS cells for each condition. Briefly, cells were washed once with PBS and scraped into 1 ml ice cold hypotonic buffer and incubated on ice for 5 min before being lysed by 10 strokes of a Dounce homogeniser. Extracts were centrifuged for 10 min at 13 000 rpm 4°C and the supernatant (cytoplasmic extract) removed. The pellet (nuclei) was washed once with hypotonic buffer before being resuspended in 100 μl of hypertonic buffer. Lysates were then sonicated in a Biodisrupter Ultra-Sonic Waterbath (Diagenode) (2 × 30 s cycles on high) to shear the genomic DNA and clarified by centrifugation at 13 000 rpm at 4°C for 10 min and the nuclear extracts collected.

Protein concentration was determined by Bradford assay and 10–20 μg of protein was loaded per well. Immunoblotting was performed using the following antibodies: HA (Clone 16B12, Covance), Flag (M2, Sigma), human HIF1α (Clone 241809, R&D systems), XIAP (2F1, Enzo), β-actin (AC-74, Sigma), K48-linked ubiquitin chains (#4289, Cell Signaling), K63-linked ubiquitin chains (#5621, Cell Signaling), ubiquitin (#3936, Cell Signaling), Ubc13 (#4919, Cell Signaling), PARP (#9542, Cell Signaling). After washing with TBS-T, membranes were incubated with secondary antibodies for 1 h at room temperature. Enhanced chemiluminescence (Pierce) was used to visualise the blots on HyBlot CL autoradiography film (Denville Scientific).

### Preparation of Histidine-tagged tandem ubiquitin binding entities (His-TUBEs)

His-TUBEs were inducibly expressed in *Escherichia coli* BL21 Rosetta (Novagen) for 4 h at 37°C. Cells were lysed by sonication in PBS supplemented with protease inhibitors, and the lysates were clarified by 20 min centrifugation at 10 000 rpm. His-TUBEs were purified with NiNTA beads (Invitrogen) and eluted with PBS 100 mM Imidazole. His-TUBES were then dialysed in 50 mM Tris pH 8.0, 150 mM NaCl using 10 kDa cut-off dialyser-cassette (Pierce). Concentration of His-TUBES was calculated by SDS-PAGE followed by coomassie staining against a known standard.

### His-TUBE pull downs

For each pull down experiment a sub-confluent 10 cm plate of mammalian culture cells was lysed in the presence of His-TUBEs (3 μM His-TUBEs, 50 mM Tris–HCl pH 8.0, 150 mM NaCl, 20 mM imidazole, 1% NP-40). Lysates were sonicated in a Biodisrupter Ultra-Sonic Waterbath (Diagenode), and clarified by centrifugation at 4°C for 10 min at 13 000 rpm.

His-TUBE complexes were incubated end-over-end for 1 h at 4°C with 20 μl packed volume Ni-NTA agarose beads. Beads were washed 3× with wash buffer (50 mM Tris–HCl pH 8.0, 150 mM NaCl, 20 mM imidazole, 1% NP-40), before being boiled in 1× SDS loading dye. Pull downs were resolved by SDS-PAGE and analysed by immunoblotting using specific antibodies.

### Cell viability assays

Cell viability was measured using Presto Blue assays, performed according to the manufacturer's protocol. Twenty four hours after seeding, cells (5000 cells/well) were pre-treated with inhibitors for 30 min before incubation at 1% O_2_. The absorbance was recorded at 570 nm after 30 min incubation of cells with Presto Blue reagent. The cell viability was expressed as a percentage relative to untreated controls.

### Caspase activity assay

Cells were seeded into 96-well plates at a density of 5000 cells/well. Twenty four hours after seeding, cells were treated as indicated and the caspase activity was determined using Caspase-Glo assay (Promega) according to the manufacturer's protocol.

### Ubiquitination assays

For ubiquitination assays, cells were lysed at room temperature under denaturing conditions (8 M urea, 50 mM Tris [pH 8.0], 300 mM NaCl, 50 mM Na_2_HPO_4_, 0.5% NP-40, 1 mM PMSF, supplemented with protease inhibitors) and ubiquitinated material was recovered by rotation with NiNTA-agarose (Invitrogen) or streptavidin-agarose (Sigma), washed 3× with lysis buffer and analysed by western blotting.

### Quantitative reverse transcription-PCR

Total RNA was isolated using the Peqgold Total RNA Isolation Kit (Peqlab) according to the manufacturer's instructions. Reverse transcription with random and oligo(dT) primers and MMLV reverse transcriptase (Quanta Biosciences) was performed on 500 ng of total RNA.

Quantitative PCR data was generated on a Rotor-Gene Q (Qiagen) using the following experimental settings: hold 50°C for 3 min; Hold 95°C 10 min; cycling (95°C for 30 s; 58°C for 30 s; 72°C for 30 s with fluorescence measurement for 45 cycles). All values were calculated relative to maximum hypoxic induction and normalised to RPL13A levels using the Pfaffl method. Each cDNA sample was assayed in duplicate and the results shown are averages derived from three to four biological repeats with error bars indicating the standard deviation.

Primers used were: RPL13A sense 5′-CCT GGA GGA GAA GAG GAA AGA GA-3′, antisense 5′-TTG AGG ACC TCT GTG TAT TTG TCA A-3′; ANKRD37 sense 5′-GTC GCC TGT CCA CTT AGC C-3′, antisense 5′-GCT GTT TGC CCG TTC TTA TTA CA-3′; CAIX sense 5′-CTT TGC CAG AGT TGA CGA GG-3′, antisense 5′-CAG CAA CTG CTC ATA GGC AC-3′; GLUT1 sense 5′-CTG GCA TCA ACG CTG TCT TC-3′, antisense 5′-GCC TAT GAG GTG CAG GGT C-3′; HIF1α sense 5′-CAT AAA GTC TGC AAC ATG GAA GGT-3′, antisense 5′-ATT TGA TGG GTG AGG AAT GGG TT-3′; HIF1β sense 5′-CAA GCC CCT TGA GAA GTC AG -3′, antisense 5′-GAG GGGCTAGGCCAC TAT TC-3′; PDK1 sense 5′-AGT TCA TGT CAC GCT GGG TA-3′, antisense 5′-CAG CTT CAG GTC TCC TTG GA-3′; VEGF sense 5′-CCT GGT GGA CAT CTT CCA GGA GTA CC-3′, antisense 5′-GAA GCT CAT CTC TCC TAT GTG CTG GC-3′.

### Chromatin immunoprecipitation (ChIP)

Proteins were cross-linked with formaldehyde for 10 min. 0.125 mM glycine was added, and cells washed with ice cold PBS. Cells were lysed with lysis buffer (1% SDS, 10 mM EDTA, 50 mM Tris–HCl, pH 8.1, 1 mM PMSF), then sonicated in a Biodisrupter Ultra-Sonic Waterbath (Diagenode) (10 × 20 s cycles on high) to shear the genomic DNA and clarified by centrifugation at 13 000 rpm at 4°C for 10 min. The supernatant was precleared with sheared salmon sperm DNA and protein G-Sepharose beads (Sigma). The supernatant was incubated with specific antibodies overnight (1 μg per IP), and then antibody protein complexes were captured with protein G-Sepharose beads for 1 h. After an extensive wash step, the complexes were eluted with buffer (100 mM NaHCO_3_, 1% SDS) and incubated with proteinase K. DNA was purified by phenol chloroform extraction. Quantative PCR was performed on the purified immunoprecipitated DNA using specific primers. ANKRD37 HRE sense 5′-AAA CTC CCG CTT CTC CGT AA-3′; ANKRD37 HRE antisense 5′-TAA GTC AGT GGG CGT GAG AG-3′; CAIX HRE sense 5′-GAC AAA CCT GTG AGA CTT TGG CTC C-3′; CAIX HRE antisense 5′-AGT GAC AGC AGC AGT TGC ACA GTG-3′.

### Immunofluorescence

For immunofluorescence, cells were grown on coverslips and treated as indicated prior to fixation by incubation in 3.7% formaldehyde/PBS for 15 min. Cells were permeabilised in PBS-1% Triton X-100–0.05% Tween for 15 min and then blocked in PBS–0.05% Tween supplemented with 10% BSA for 30 min. HIF1α antibody was diluted 1:100 in 1% BSA-0.5% Tween20–PBS incubated for 1 h. Secondary antibody labelled with X-Red was purchased from Jackson Immunoresearch and used at 1:500. Cover slips were mounted using Pro Long Gold mounting media.

## RESULTS

### HIF1α is ubiquitinated by XIAP

**Figure 1.**
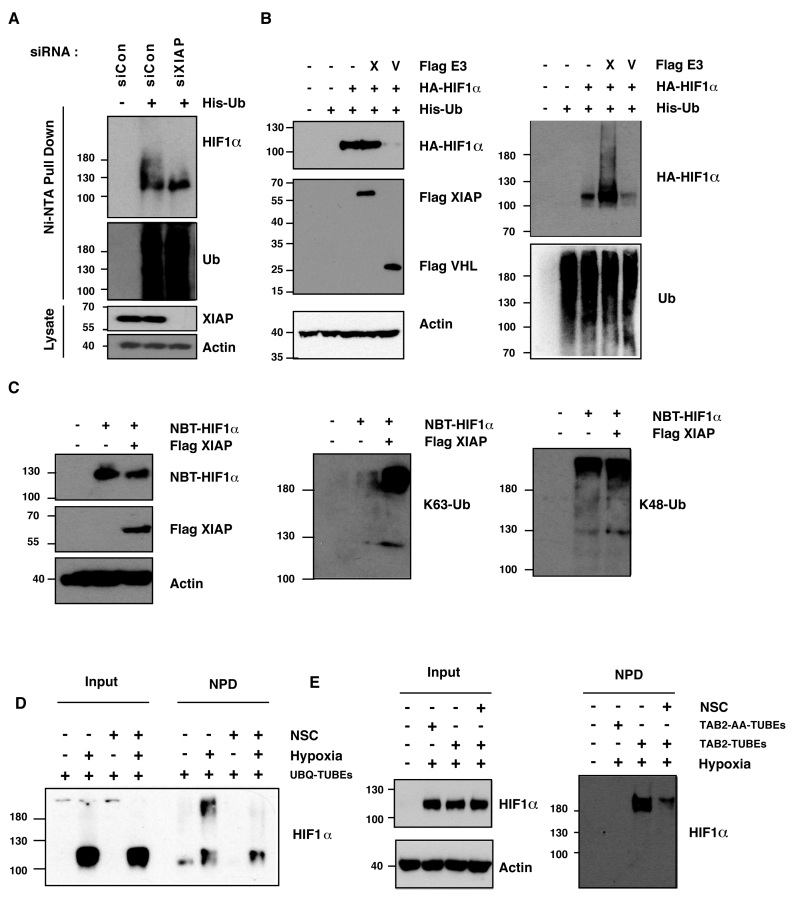
XIAP ubiquitinates HIF1α. (**A**) U2OS cells were transfected with siRNA oligonucleotides and His-tagged ubiquitin as indicated. Lysates were prepared and precipitated using nickel-conjugated beads followed by western blotting using the indicated antibodies. (**B**) HA-tagged plasmid encoding HIF1α was coexpressed in HEK293 cells with His-tagged ubiquitin (Ub), FLAG-XIAP (X) or FLAG-VHL (V) as indicated. Lysates were prepared 48 h post-transfection and ubiquitinated material was recovered by incubation with nickel-conjugated beads and analysed by western blotting. (**C**) Biotinylated-HIF1α was coexpressed in HEK293 cells with FLAG-tagged XIAP. HIF1α was recovered from lysates by incubation with streptavidin-coated beads and analysed using antibodies directed against specific ubiquitin chains. (**D**) U2OS cells were pre-treated with 2μM NSC697923 for 30 min before being exposed to 1% O_2_ for 3 h. Cells were lysed in the presence of UBQ-TUBEs. Ubiquitin conjugates were purified using NiNTA agarose beads and analysed by immunoblotting using the indicated antibodies. (**E**) U2OS cells were pre-treated with 2 μM NSC697923 for 30 min before being exposed to 1% O_2_ for 3 h. Cells were lysed in the presence of TAB2-TUBEs or TAB2-AA-TUBEs as indicated. Proteins conjugated to Lys^63^-linked ubiquitin chains were purified using Ni-NTA agarose beads and analysed by immunoblot.

Control of HIF is primarily mediated by modulating the accumulation and activity of the HIF1α subunit (1,2). HIF1α levels are predominantly regulated by Lys^48^-linked polyubiquitination by VHL, however alternative E3 ligases can modulate the levels and activity of HIF1α independently of the well-described VHL-dependent pathway (2). The X-linked inhibitor of apoptosis (XIAP) belongs to the family of IAP (inhibitor of apoptosis) proteins, and its best-described role is as an inhibitor of caspases-3, -7 and -9 (10). However, in addition to its cytoprotective role, XIAP also acts as an E3 ubiquitin ligase by virtue of its carboxy-terminal RING domain (10). XIAP has been described as a key signaling intermediate required for receptor activated canonical and non-canonical NF-κB (11), regulation of cell motility (10), activation of MAPK/JNK (12), Wnt signaling (13) and hypoxia-induced NF-κB (14). We therefore investigated if XIAP might also function as an alternative HIF1α E3 ubiquitin ligase that contributes to the HIF1-dependent response to hypoxic stress. Ubiquitin pull down assays reveal that XIAP depletion by siRNA resulted in impaired HIF1α ubiquitination compared to cells transfected with a non-targeting control (Figure 1A). To investigate whether overexpression of XIAP altered HIF1α ubiquitination HEK293 cells were co-transfected with plasmids encoding HIF1α and His-tagged ubiquitin, in the presence or absence of exogenous XIAP. When HIF1α was expressed alone, polyubiquitination was observed, consistent with HIF1α being a ubiquitinated protein (Figure 1B). The levels of ubiquitin-modified HIF1α isolated in an ubiquitin pull down were markedly increased with cotransfection of XIAP with no decrease in the levels of the HIF1α protein being observed (Figure 1B). Co-expression of the best-described HIF1α ubiquitin ligase VHL reduced total levels of HIF-1α, consistent with its known role in promoting HIF1α proteasomal degradation. Polyubiquitination of HIF1α was specific since XIAP did not promote the conjugation of ubiquitin chains to other regulators of the HIF pathway; PHD1, PHD2, PHD3 or VHL (Supplementary Figure S1A).

### XIAP promotes the conjugation of Lys^63^-linked ubiquitin chains to HIF1α

XIAP can promote the formation both Lys^63^- and Lys^48^-linked polyubiquitin chains depending on the substrate and the E2 ubiquitin-conjugating enzyme (10). We therefore investigated which of these forms of ubiquitin chains were conjugated to HIF1α by XIAP. A plasmid encoding biotin-tagged HIF1α was transfected into HEK293 cells, together with an expression vector encoding XIAP. HIF1α conjugates were isolated by streptavidin pull down and subjected to immunoblot analysis using antibodies designed to recognise specific ubiquitin chain linkages (Figure 1C). Lys^48^-linked ubiquitin chains were observed conjugated to HIF1α, but no increase in the levels of these were seen when XIAP was overexpressed (Figure 1C). In contrast, levels of Lys^63^-linked ubiquitin chains associated with HIF1α were markedly increased in cells expressing exogenous XIAP (Figure 1C). The data indicate that XIAP promotes the formation of the non-degradative Lys^63^-linked ubiquitin chains onto HIF1α, but does not affect the formation of Lys^48^-linked chains.

### Ubc13 activity is required for HIF1α Lys^63^ polyubiquitination

The formation of Lys^63^-linked ubiquitin chains is dependent on Ubc13, the active subunit of an E2 ubiquitin conjugating enzyme complex (15). Cells were treated with two independent small molecule inhibitors of Ubc13, NSC697923 or BAY 11–7082, to investigate whether chemical inhibition of Ubc13 could prevent Lys^63^-ubiquitination of HIF1α (16,17). Levels of Lys^63^-linked ubiquitin chains associated with biotinylated HIF1α were markedly decreased in cells treated with NSC697923 or BAY 11-7082, however total levels of HIF1α remained constant (Supplementary Figure S1B). These data indicate that HIF1α is modified by Lys^63^-linked ubiquitin chains in a manner dependent on Ubc13 activity.

To investigate whether Ubc13 inhibition could alter the ubiquitin status of endogenous HIF1α we isolated ubiquitin conjugates from hypoxic cells using tandem ubiquitin binding entities based on the ubiquitin binding domain of the ubiquilin protein (UBQ-TUBEs). UBQ-TUBEs bind with high affinity to Lys^63^- or Lys^48^-linked polyubiquitin chains (18) and efficiently isolate ubiquitinated HIF1α for U2OS cells treated with proteasomal inhibitor, MG132 (Supplementary Figure S1C). Hypoxic U2OS cells were pretreated with the Ubc13 inhibitor, NSC697923, then lysed in the presence of UBQ-TUBEs (18). Cells treated with Ubc13 inhibitor had less ubiquitinated HIF1α as determined immunoblot following by TUBE pull down, indicating that the total ubiquitinated pool of endogenous HIF1α in hypoxic conditions was reduced when Ubc13 activity was inhibited (Figure 1D).

To examine levels of endogenous Lys^63^-linked ubiquitination of HIF1α we prepared TUBEs based on the ubiquitin-binding domain of TAB2 (TAB2-TUBEs) and used these to isolate Lys^63^-linked ubiquitin conjugates from cells. The UBD of TAB2 binds specifically and efficiently to Lys^63^-linked ubiquitin chains and TAB2-TUBEs efficiently isolated Lys^63^-linked ubiquitin conjugates from cells, as determined by immunoblot with a Lys^63^-linked ubiquitin chain specific antibody (Supplementary Figure S1D). The binding of ubiquitin to the TAB2-TUBEs was specific as pull down performed with TAB2 TUBES with point mutations (T674A F675A) that disrupt the ubiquitin interaction domain (TAB2-AA-TUBEs) did not purify any Lys^63^-linked ubiquitinated proteins (Supplementary Figure S1D). HIF1α was specifically isolated by TAB2-TUBE purification from hypoxic cells, but not with TAB2-AA-TUBEs, consistent with HIF1α being a Lys^63^-ubiquitinated protein (Supplemental Figure S1D). Cells treated with Ubc13 inhibitor had less Lys^63^-ubiquitinated HIF1α as determined by TAB2-TUBE pull down, indicating that Ubc13 is required for Lys^63^-dependent ubiquitination of HIF1α (Figure 1E). To determine if these effects were due to Lys^63^-dependent ubiquitination impacting on the VHL-dependent HIF1 degradation pathway UBQ-TUBE pull downs were performed on VHL-deficient RCC4 cells which have constitutively high levels of HIF1α. Levels of ubiquitinated HIF1α are suppressed by Ubc13 inhibition in the absence of VHL, indicating that this is a distinct parallel regulatory pathway (Supplementary Figure S1E). Together these data indicate that HIF1α is conjugated to Lys^63^-linked ubiquitin chains in cells and that XIAP and Ubc13 are important for the polyubiquitination of endogenous HIF1α.

### Lys^63^-linked ubiquitination of HIF by XIAP/Ubc13 promotes expression of hypoxia-induced gene products

**Figure 2.**
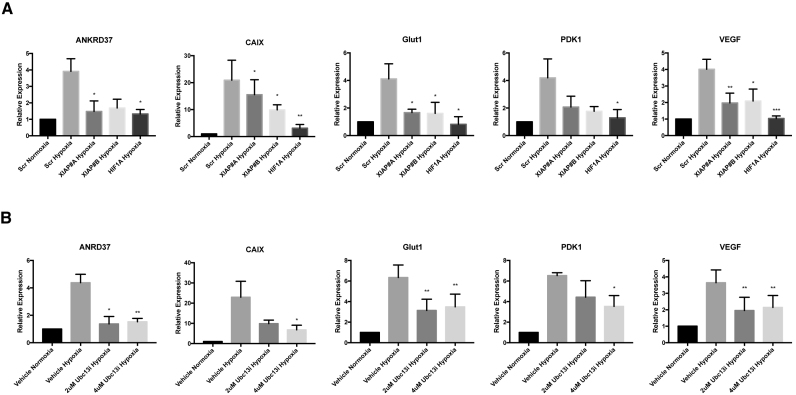
Inhibition of Lys^63^-linked ubiquitination of HIF1α suppresses HIF1-dependent gene expression. (**A**) Quantitative RT–PCR analysis of CAIX, ANKRD37, Glut1, PDK1 and VEGF mRNA prepared from U2OS cells expressing siRNAs targeting XIAP, HIF1α or a control siRNA, and subsequently exposed to 1% O_2_ for 24 h. All values are normalised to RPL13A mRNA and fold change calculated from control samples prepared in normoxic conditions. (**B**) Quantitative RT–PCR analysis of CAIX, ANKRD37, Glut1, PDK1 and VEGF mRNA prepared from U2OS cells pre-treated with the indicated concentrations of NSC697923 for 30 min before being exposed to 1% O2 for 7 h. All values are normalised to RPL13A mRNA and fold change calculated from normoxic controls. Significance calculated relative to hypoxic controls using a one-way ANOVA using the Dunnett multiple comparison test.

To examine the role of XIAP-mediated ubiquitination of HIF1α in the expression of hypoxia-responsive target genes, we performed quantitative real time PCR analysis of the expression of HIF1 target genes in cells depleted of XIAP. U2OS cells transiently transfected with either control siRNAs or siRNAs designed to reduce XIAP expression were subjected to hypoxia for the indicated time points (Figure 2A). RNA was isolated from these cells and was used to quantitate the transcript levels of the hypoxia-responsive genes; CAIX, ANKRD37, GLUT1, PDK1 and VEGF. As expected, exposure to low oxygen resulted in an increase in expression of all of these genes, as compared to control (Figure 2A). In cells transfected with siRNA to deplete XIAP the levels of HIF target genes were reduced, although not to the extent we observed with siRNAs depleting HIF1α mRNA. These data are consistent with XIAP modulating the expression HIF-dependent genes (Figure 2A).

The importance of Lys^63^-linked ubquitination for the expression of HIF1-dependent gene expression examined by using specific chemical inhibitors of Ubc13. Pre-treatment of U2OS cells with NSC697923 suppressed the hypoxia-induced expression of each of the HIF-target genes, consistent with Ubc13 activity being important for the expression of HIF-target genes (Figure 2B). Similar results were observed using a second inhibitor of the Lys^63^-linked ubiquitination pathway, BAY 11–7082 (Supplementary Figure S2A). Experiments were then performed using siRNA to deplete Ubc13 levels. Ubc13 siRNA efficiently reduced levels of Ubc13 protein in U2OS cells as measured by western blot (Supplementary Figure S2B). Depletion of Ubc13 resulted in a decrease in the expression of hypoxia-induced HIF target genes (Supplementary Figure S2C). HEK293 and PC-3 cells pretreated with either NSC697923 or BAY 11-7082 also had reduced hypoxia-induced HIF-dependent gene expression, indicating that the Lys^63^-linked ubiquitination pathway is important for the HIF-dependent response in different cell lines (Supplementary Figure S2D).

### HIF1α protein and transcript levels are not altered by Ubc13-dependent Lys^63^-linked ubiquitination

Lys^63^-linked polyubiquitination is necessary for the activation of the NF-κB family of transcription factors in response to several stimuli (19,20). Indeed, both NSC697923 and BAY 11-7082 have been shown to prevent NF-κB activation in numerous cell lines through inhibition of Ubc13-dependent Lys^63^-linked ubiquitination of the components of the NF-κB signal transduction cascade (16,17,21). As both HIF1α and HIF1β are direct NF-κB target genes (22–24) we investigated whether Ubc13 inhibition was altering HIF activity by altering the levels of HIF1α or HIF1β mRNA. HIF1α and HIF1β mRNA levels were analysed by quantitative RT-PCR (qRT-PCR), using RNA purified from U2OS cells treated with NSC697923 or BAY 11-7082 (Supplementary Figures S3A and S3B). Inhibition of Ubc13 revealed no significant differences in either HIF1α or HIF1β mRNA levels, indicating that the role of Lys^63^-linked polyubiquitin in regulating HIF is not due to an effect on HIF1α or HIF1β mRNA production or stability, but most likely Ubc13 inhibitors exert their effects on the HIF pathway post-transcriptionally (Supplementary Figures S3A and S3B).

**Figure 3.**
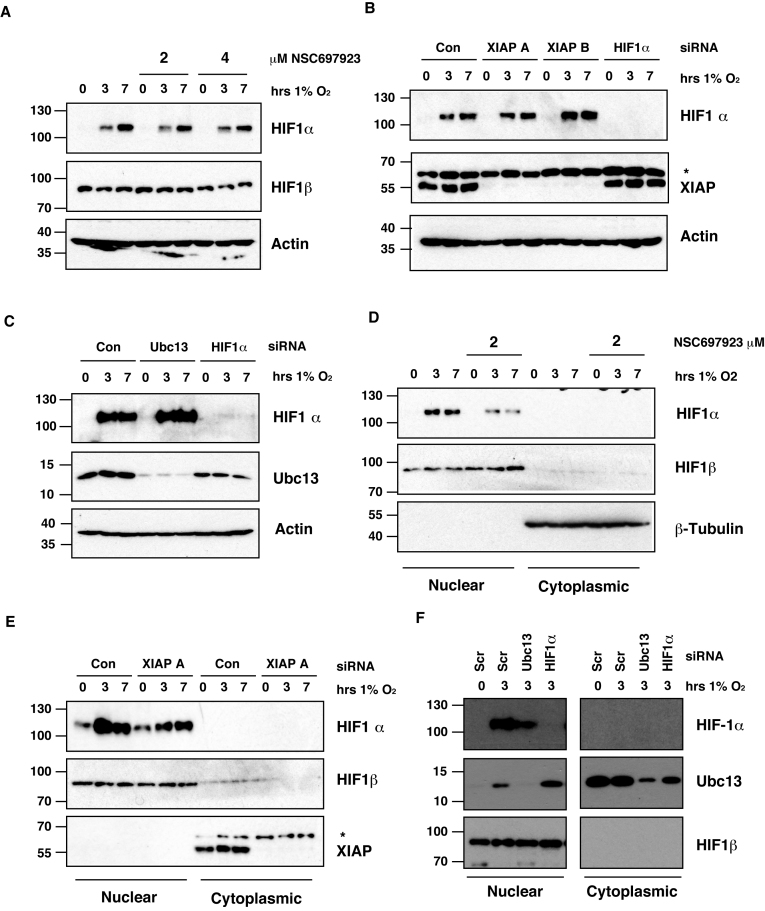
Suppression of Lys^63^-linked polyubiquitination of HIF1α reduces HIF1α nuclear localisation. (**A**) Whole cell lysates were prepared from U2OS cells pre-treated with the indicated concentrations of NSC697923 for 30min before being exposed to 1% O2 for 3 and 7 h. Lysates were subjected to immunoblot analysis to assess expression levels of the indicated proteins. (**B** and **C**) U2OS cells transfected with the indicated siRNAs before being exposed to 1% O_2_ for the indicated times. Whole-cell lysates (WCLs) prepared from these cells were subjected to immunoblot analysis to assess expression levels of the indicated proteins. (**D**) Cytoplasmic and nuclear extracts were prepared from U2OS cells pre-treated with 2 μM NSC697923 for 30 min before being exposed to 1% O_2_ for indicated times. Extracts were analysed by immunoblotting using the indicated antibodies. (**E** and **F**) Cytoplasmic and nuclear extracts were prepared from U2OS cells transfected with the indicated siRNAs before being exposed to 1% O_2_ for the indicated time points.

The primary mechanism for controlling HIF1 activity is through the protein levels of the HIF1α subunit (2). To investigate whether Ubc13 inhibition was altering the protein levels of HIF1α following hypoxia, U2OS cells were pre-treated with NSC697923 and incubated at low oxygen for the indicated time points (Figure 3A). No significant decrease in the oxygen-dependent accumulation of HIF1α, or total levels of HIF1β was observed after Ubc13 inhibition in hypoxia (Figure 3A and Supplementary Figures S3A and S3B).

We then examined whether XIAP or Ubc13 depletion altered the total levels of HIF1α following hypoxic stress in U2OS cells transfected with siRNAs targeting XIAP or Ubc13 and exposed to hypoxia for the indicated times. No change was observed in the levels of HIF1α stabilised by hypoxic stress in the presence or absence of XIAP or Ubc13 (Figure 3B and C). These results suggest that knockdown of XIAP or chemical inhibition / depletion of Ubc13 does not alter the HIF1 transcriptional response by altering total levels of HIF1α stabilised by low oxygen.

### Lys^63^-linked ubiquitination controls HIF1α subcellular localisation in hypoxia

To explore how Lys^63^-linked polyubiquitination alters the activity of HIF we performed subcellular fractionation experiments to investigate whether Ubc13 inhibition alters the subcellular localisation of stabilised HIF1α in hypoxic cells. Nuclear and cytoplasmic extracts were prepared from U2OS cells pre-treated with NSC697923 following hypoxic stress and immunoblotted for HIF1α levels (Figure 3D). HIF1α was observed in the nuclear fraction in low oxygen conditions, however, the levels of nuclear HIF1α are lower in cells treated with NSC697923 (Figure 3D). No effect was seen on the related HIF family member HIF1β. We did not observe an increase of HIF1α in the cytoplasmic fraction in these experiments as a result of HIF1α concentration being below the level of detection. To enable detection, HIF1α was immunoprecipitated from cytoplasmic extracts and subsequently immunoblotted. An increase in cytoplasmic HIF1α was observed in hypoxic cells treated with NSC697923, consistent with the decrease of HIF1α in the nucleus (Supplementary Figure S4A). In addition, we examined HIF1α levels in cells transfected with siRNA to deplete Ubc13 or XIAP protein levels. A decrease in the levels of nuclear HIF1α was detected in the absence of Ubc13 (Figure 3E and F). Interestingly, the data reveal that a proportion of Ubc13 also translocates to the nucleus in hypoxic conditions, suggesting a nuclear role for Ubc13 in hypoxic cells (Figure 3F). To complement biochemical subcellular fractionation experiments, HIF1α localisation was measured by immunofluorescence following hypoxic stress. U2OS cells pre-treated with NSC697923 and exposed to hypoxia for 3 hours were fixed and stained using a specific HIF1α antibody. In hypoxic U2OS cells, HIF1α is readily observed in the nucleus as confirmed by co-localisation with a nuclear stain (Supplementary Figure S4B). In cells, pre-treated with NSC697923 a dose dependent reduction in the nuclear levels of HIF1α was observed, consistent with biochemical subcellular fractionation data (Supplementary Figure S4B).

### Ubc13 inhibition impairs HIF1α promoter occupancy of HIF target genes

**Figure 4.**
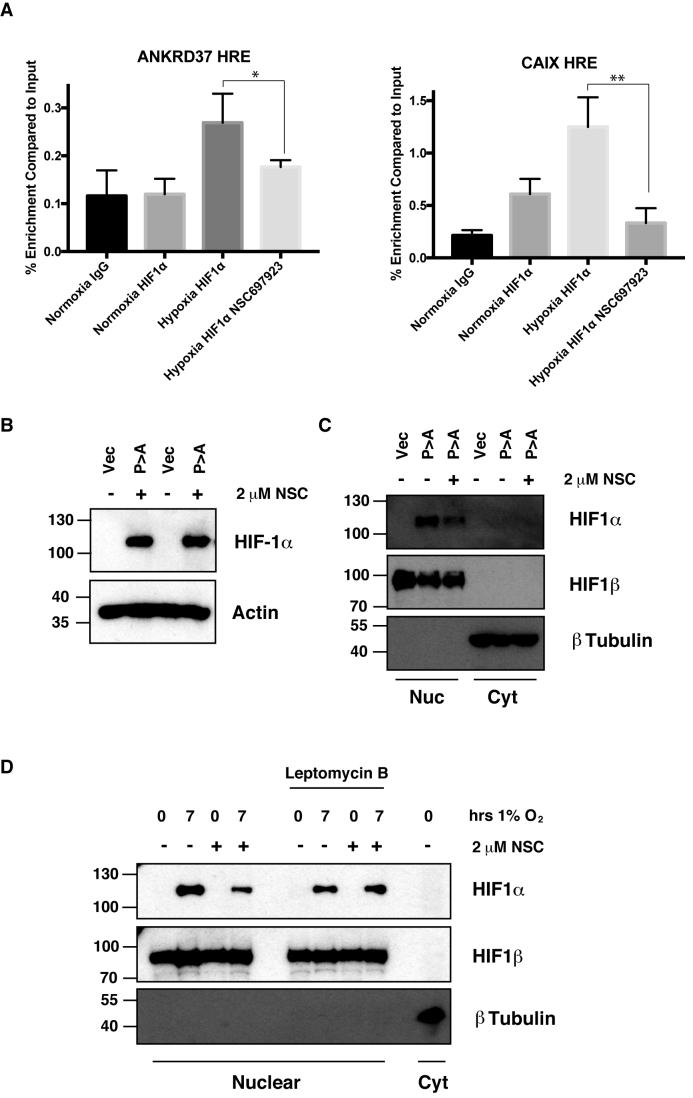
Ubc13 activity is necessary for HIF1α nuclear retention. (**A**) ChIP assays using Control IgG and HIF1α antibodies were performed on cell extracts from U2OS cells pre-treated with 2μM NSC697923 for 30 min before being exposed to 1% O2 for 3 h. Immunoprecipitated DNA was analysed by qPCR using the indicated primer pairs and normalised to input genomic DNA. (**B** and **C**) U2OS cells were transfected with HA-HIF1α (P402A, P564A) or empty vector and treated with 2 μM NSC697923 for 3 h. Whole cell lysates (B) or nuclear and cytoplasmic extracts (C) were prepared. Extracts were analysed by immunoblotting using the indicated antibodies. (**D**) Cytoplasmic and nuclear extracts were prepared from U2OS cells treated with 2 μM NSC697923 and 5 ng/ml Leptomycin B as indicated before being exposed to 1% O_2_ for 3 h. *P*-values are significant according to the Student's *t*-test; *P < 0.05, **P < 0.01.

Our data reveals that inhibition of Lys^63^-linked polyubiquitination of HIF1α alters the transactivation of HIF target genes, but does not alter total levels of HIF1α accumulation in low oxygen conditions. To understand how disruption to the Lys^63^-linked polyubiquitin pathway alters HIF target gene expression we examined the levels of HIF1α associated with the promoters of target genes in the presence of NSC697923. Chromatin immunoprecipitation (ChIP) assays were used to examine the occupancy of HIF1α present at the promoters of HIF target genes ANKRD37 and CAIX in hypoxic U2OS cells (Figure 4A). A reduction in the levels of HIF1α associated with the promoters of HIF target genes was observed in hypoxic cells pre-treated with the NSC697923 (Figure 4A). These data suggest that inhibition of Lys^63^-linked polyubiquitin compromises gene occupancy by HIF1α, despite the total cellular levels of HIF1α being unaltered (Figure 4A).

### Ubc13 activity is necessary for HIF1α nuclear retention

Movement of transcription factors to and from the nuclear compartment depends on the presence of targeting sequences in the protein, which are recognised by specific nuclear transport receptors and nuclear export proteins. Transcription factors, such as HIF1α, can be regulated by control of their nuclear import, export or a combination of both (25).

To investigate, mechanistically, how HIF1α nuclear localisation is altered we tested whether Ubc13 inhibition could promote the export of constitutively stabilised, nuclear HIF1α, where the VHL binding sites, proline 402 and 564, have been mutated to alanine (HIF1α P>A). As expected HIF1α P>A was efficiently expressed and was predominantly found in the nucleus of transfected cells (Figure 4B and C). Cells treated with the Ubc13 inhibitor NSC697923 has less nuclear HIF1α P>A than untreated controls, despite the total level of the exogenous protein remaining unchanged. These data indicate that Lys^63^-linked polyubiquitination-dependent nuclear retention of HIF1α does not result from effects on VHL mediated HIF1α degradation (Figure 4B and C).

Export of HIF1α from the nucleus is mediated by the major nuclear export pathway dependent on the exportin protein, Crm1 (26). To examine the role of Lys^63^-linked polyubiquitination in the export of HIF1α the well-described Crm1 inhibitor, leptomycin B, was used (27). Nuclear extracts were prepared from hypoxic U2OS cells pre-treated with NSC697923 and leptomycin B as indicated (Figure 4D). Hypoxic U2OS cells treated with NSC697923 had decreased levels of nuclear HIF1α, consistent with previous observations. However, in cells pre-treated with leptomycin B the levels of nuclear HIF1α induced by hypoxic stress were insensitive to inhibition of the Lys^63^-linked polyubiquitin pathway (Figure 4D, Supplementary Figure S4D). Lys^63^-linked polyubiquitin of HIF1α therefore acts to prevent its nuclear export rather then being required for nuclear import.

### Ubc13 inhibition sensitises cells to hypoxic stress

**Figure 5.**
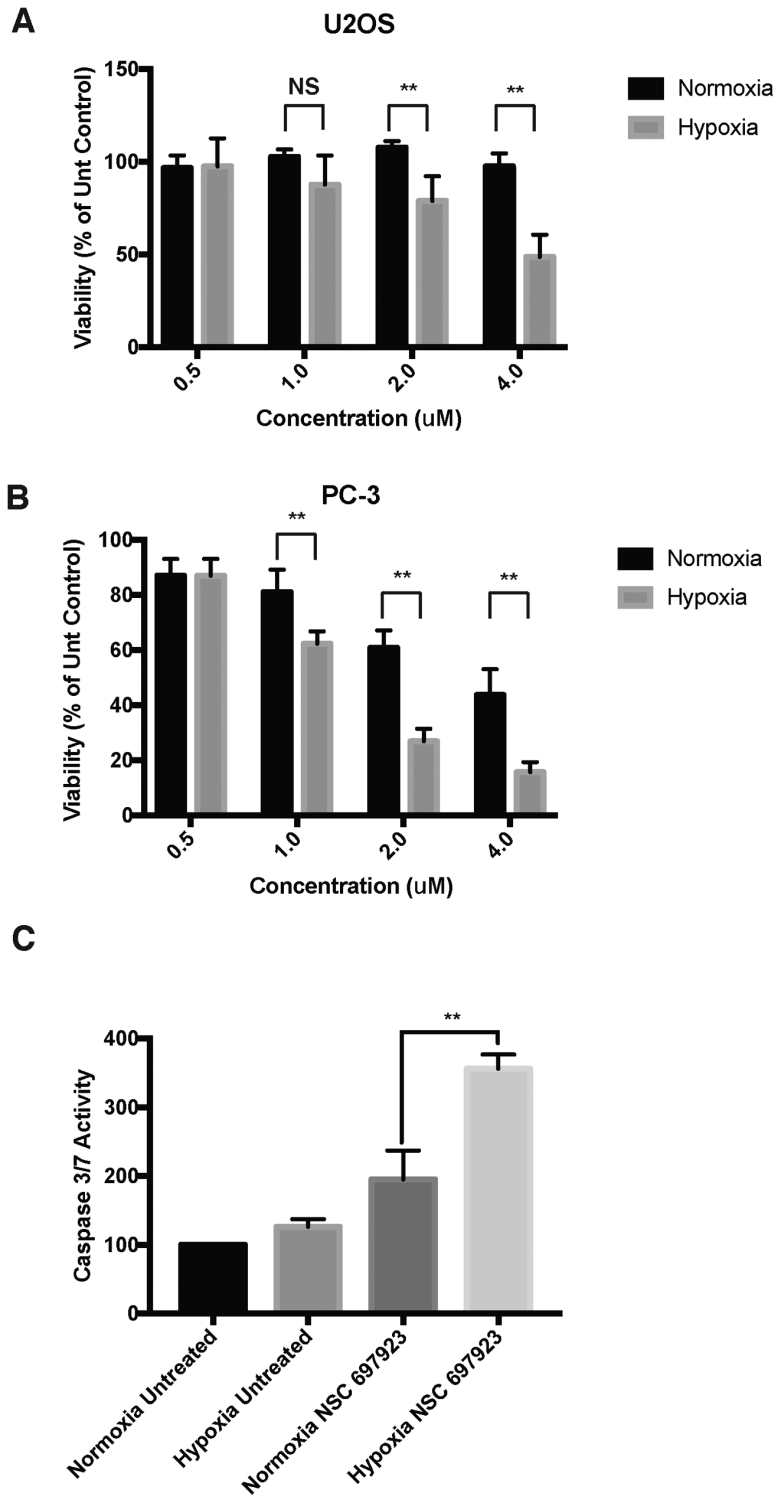
Hypoxic cells are more sensitive to Ubc13 inhibitors. (**A**) U2OS or (**B**) PC-3 cells were pretreated with the indicated concentrations of NSC697923 before being exposed to 1% O_2_ for 24 h. Cell viability was measured by Presto Blue assay and values normalised to untreated controls. (**C**) U2OS treated as in with 2uM NSC697923 (A). Caspase activity was determined by Caspase-Glo assay and values normalised to normoxic controls. Error bars represent standard deviation. *P*-values are significant according to the Student's *t*-test; *P < 0.05, **P < 0.01.

As the HIF pathway is an attractive target for numerous human disease states we investigated whether Ubc13 inhibition could alter viability of hypoxic cells. We therefore titrated the Ubc13 inhibitor, NSC697923, into U2OS and PC-3 cells and investigated their viability after 24 h in either normoxic or hypoxic conditions. Cells cultured in hypoxic conditions had increased sensitivity to NSC697923, with a decrease in the viability observed at much lower concentrations of the Ubc13 inhibitor (Figure 5A and B). To determine whether changes in the levels of apoptosis were responsible for the loss of viability in hypoxic cells treated with the Ubc13 inhibitor and measured the activity of caspases-3/7, the executioner caspases activated in apoptotic cell death. Hypoxic cell treated with NSC697923 had increased caspase activity compared to untreated hypoxic cells, indicating a higher level of apoptosis (Figure 5C). Western blots were performed to examine the levels of the caspase-3 target, PARP. The data indicate higher levels of PARP cleavage in hypoxic cells treated with NSC697923 consistent with higher levels of active caspase-3 (Supplemental Figure S5). These data indicate that the conjugation of Lys^63^-linked polyubiquitin chains is important for the adaptive cellular response to hypoxia.

**Figure 6.**
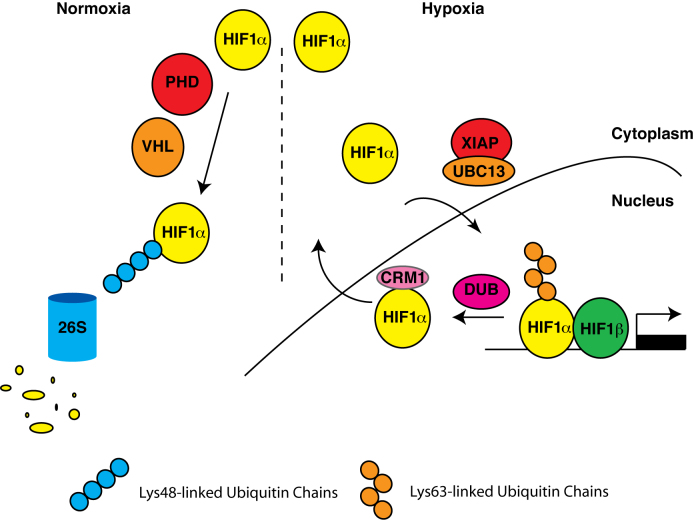
Model of HIF1α regulation by XIAP and Ubc13.

Our data collectively reveal that the formation of Lys^63^-linked ubiquitin chains to HIF1α promotes the full expression of HIF target gene expression in hypoxic cells (Figure 6). Conjugation of Lys^63^-linked ubiquitin chains to HIF1α by XIAP controls HIF1 activity, not by altering its stability, but by controlling nuclear export of HIF1α in a Crm1 dependent manner therefore controlling its ability to drive the expression of target genes.

## DISCUSSION

The results of the present study indicate that the full activation of the HIF-dependent transcriptional response requires the action of E3 ubiquitin ligases that conjugate Lys^63^-ubiquitin chains to HIF1α. Our data, using small-molecule inhibitors and specific siRNAs targeting the ubiquitin-conjugating enzyme, Ubc13, demonstrate that the hypoxia-dependent expression of HIF1α target genes is suppressed when the Lys^63^-ubiquitin conjugation pathway is disrupted.

We have identified XIAP as a key ubiquitin E3 ligase responsible for Lys^63^-linked ubiquitination of HIF1α. XIAP-dependent conjugation of Lys^63^-linked ubiquitin chains correlates with elevated HIF1α activity and like inhibition of Ubc13 alters HIF1α subcellular localisation. Our data suggest that XIAP is involved in a parallel pathway responsible for controlling the level of the HIF1 dependent hypoxic response. XIAP, a well-described suppressor of cell death has also been shown to be highly overexpressed in many forms of cancer. Both HIF1α and XIAP expression have been correlated with the progression or severity of neoplastic disease. A longitudinal study of patients with acute myeloid leukemia (AML) revealed a correlation between poor prognosis and elevated XIAP levels (28). Interestingly, other studies have identified a correlation between HIF1α levels and poor prognosis in AML, perhaps providing indirect evidence of a link between these proteins contributing to disease (29).

Multiple roles for Lys^63^-linked polyubiquitylation in immune responses have emerged in recent years, including regulation of NF-κB, TGFβ and MAPK signaling, indicating the importance of this regulatory strategy (30). Our data indicates that Ubc13 plays a critical role in the HIF pathway, which also has crucial roles in the immune response (31). Intriguingly, data obtained in experiments in Ubc13 conditional knockout mice revealed that Ubc13 as a crucial regulator of hematopoiesis, a developmental process in which HIF signaling has a critical role (32,33).

Inhibition of the formation of Lys^63^-linked ubiquitin chains on HIF1α causes a decrease in HIF target gene expression in part due to preventing nuclear retention of HIF1α and thus blocking its association with HIF target genes. Our data indicates that inhibiting Lys^63^-linked polyubiquitination of HIF1α accelerates the nuclear export of HIF1α in a Crm1-dependent manner. The control of nuclear localisation of proteins by non-degradative polyubiquitination has been previously described and our data may indicate a conserved mechanism through which this post-translational modification can alter the subcellular localisation of protein substrates (34,35). *In silico* prediction of ubiquitination sites on the HIF1α protein reveals that the four lysine residues (Lys^625^, Lys^629^, Lys^636^ and Lys^649^) surrounding, or within, the atypical Crm1-dependent nuclear export sequence of HIF1α are likely to be modified by ubiquitin to a medium or high confidence interval (26) (http://www.ubpred.org/). The addition of ubiquitin chains to or near the nuclear export sequence may prevent the interaction with Crm1, and suggest that removal of these chains by an unknown deubiquitinating (DUB) enzyme may be required to facilitate HIF1α nuclear export (Figure 6). Intriguingly, our subcellular fractionation studies also reveal that Ubc13 enters the nuclear compartment under conditions of hypoxic stress where it could potentially target multiple factors. XIAP does not move to the nucleus in hypoxia, however subcellular fractionation experiments performed in the presecnce of the nuclear export inhibitor leptomycin B, demonstrate that a proportion of the protein shuttles between the nucleus and cytoplasm, indicating a nuclear role for XIAP (Supplementary Figure S4C).

Our findings indicate that hypoxic cells are sensitive to Ubc13 inhibition. This suggests that the Lys^63^-linked ubiquitination pathway is important for the cellular response to hypoxia, either purely through targeting HIF1α, or by targeting multiple proteins in this stress response. Studies from the yeast, *Saccharomyces cerevisiae*, suggest that Lys^63^-linked ubiquitination is critical in the oxidation-dependent signaling response, perhaps indicating a conserved role for Lys^63^-linked ubiquitination being a key post translational modification in stress response pathways (36).

The data demonstrating the requirement of Ubc13 activity in the HIF-dependent hypoxic response indicates that targeting the Lys^63^-linked ubiquitination pathway may represent a potential therapeutic target in clinical pathologies with aberrant HIF activity. Indeed, there is great interest in targeting the components of the ubiquitin conjugation pathway in disease and chemical inhibitors of the proteasomal and non-degradative ubiquitin pathways are being developed as potential therapeutics (37).

Our results suggest that Ubc13 inhibition and knockdown have a more pronounced effect on the expression of HIF target gene expression suggesting that alternative E3 ubiquitin ligases also target HIF1α Lys^63^-ubiquitination. Indeed, both TRAF6 and STUB1 have been previously demonstrated to conjugate Lys^63^-linked ubiquitin chains to the HIF1α protein (38,39). Collectively, these data provide further mechanistic understanding into the regulation of the HIF-dependent transcriptional response to hypoxia. Insights into how this important transcription factor is regulated advance our knowledge into how to target this signaling pathway in disease.

## Supplementary Material

Supplementary Data
