# Supplementary material for: XIAP upregulates expression of HIF target genes by targeting HIF1α for Lys63-linked polyubiquitination
Source: Nucleic Acids Res. 2017 Jun 28;45(16):9336–47. doi: 10.1093/nar/gkx549 (PMC5766203; doi:10.1093/nar/gkx549)
Supplement: Supplementary Data [file gkx549_supp.pdf]

Supplementary Figure S1

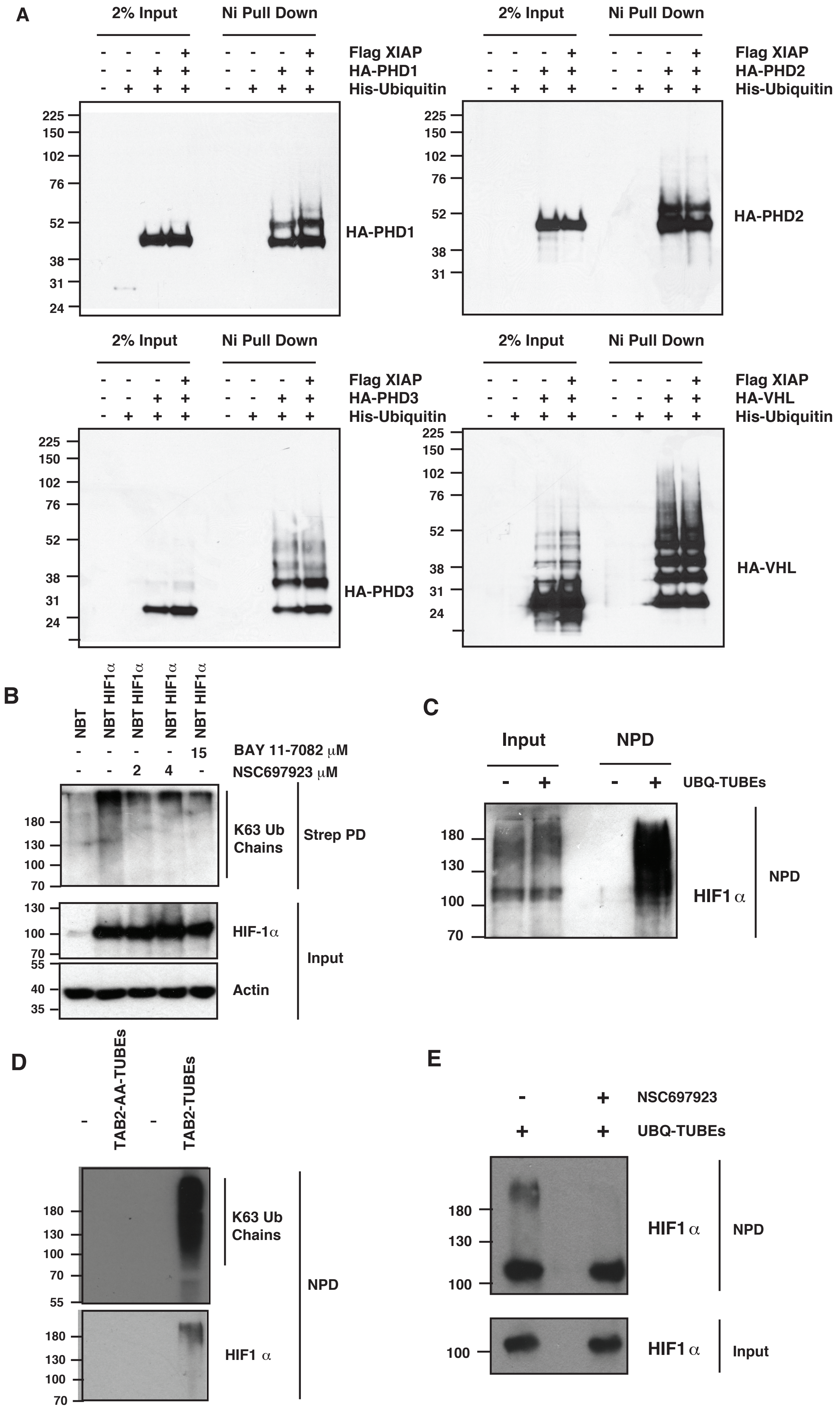

Supplementary Figure S2

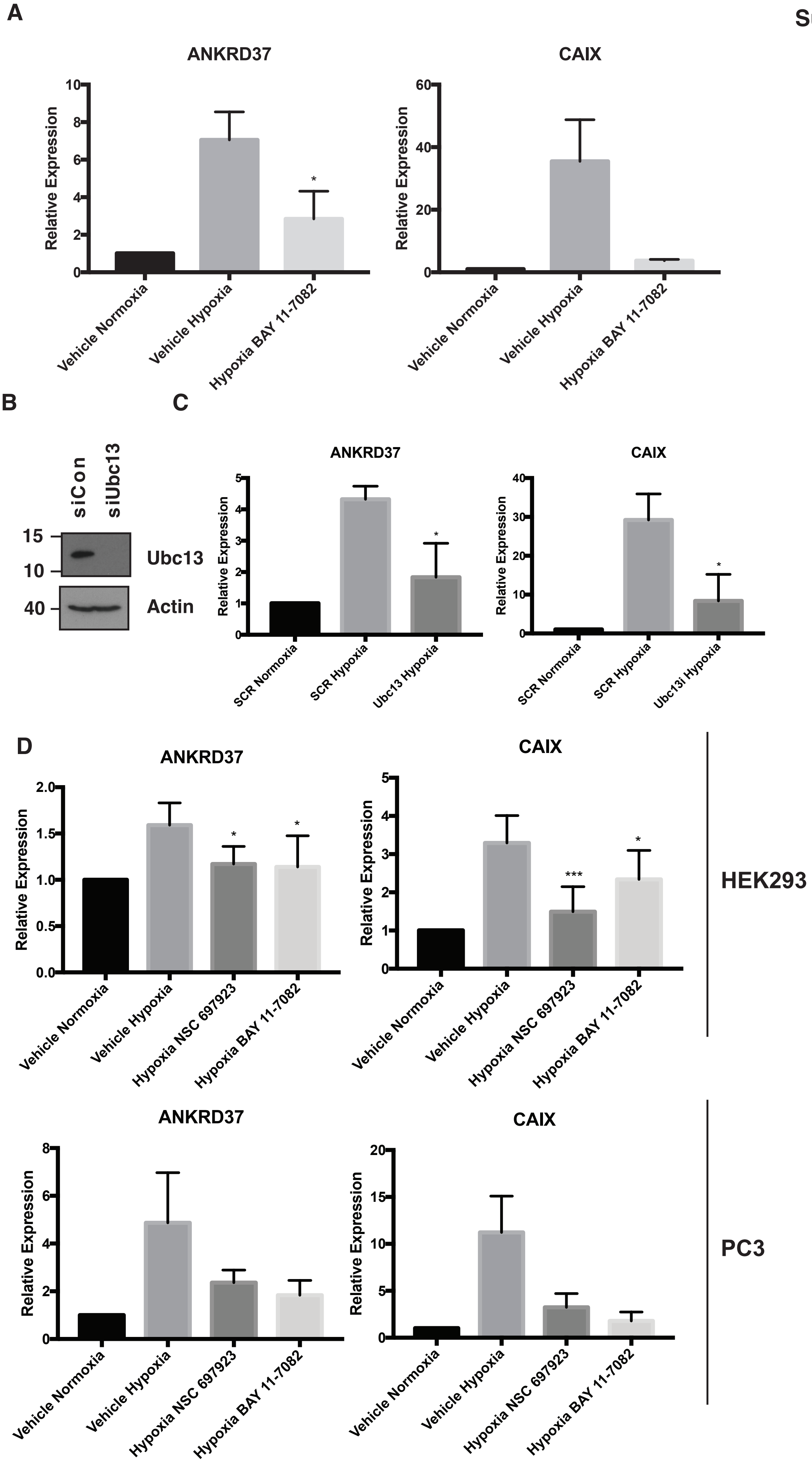

Supplementary Figure S3

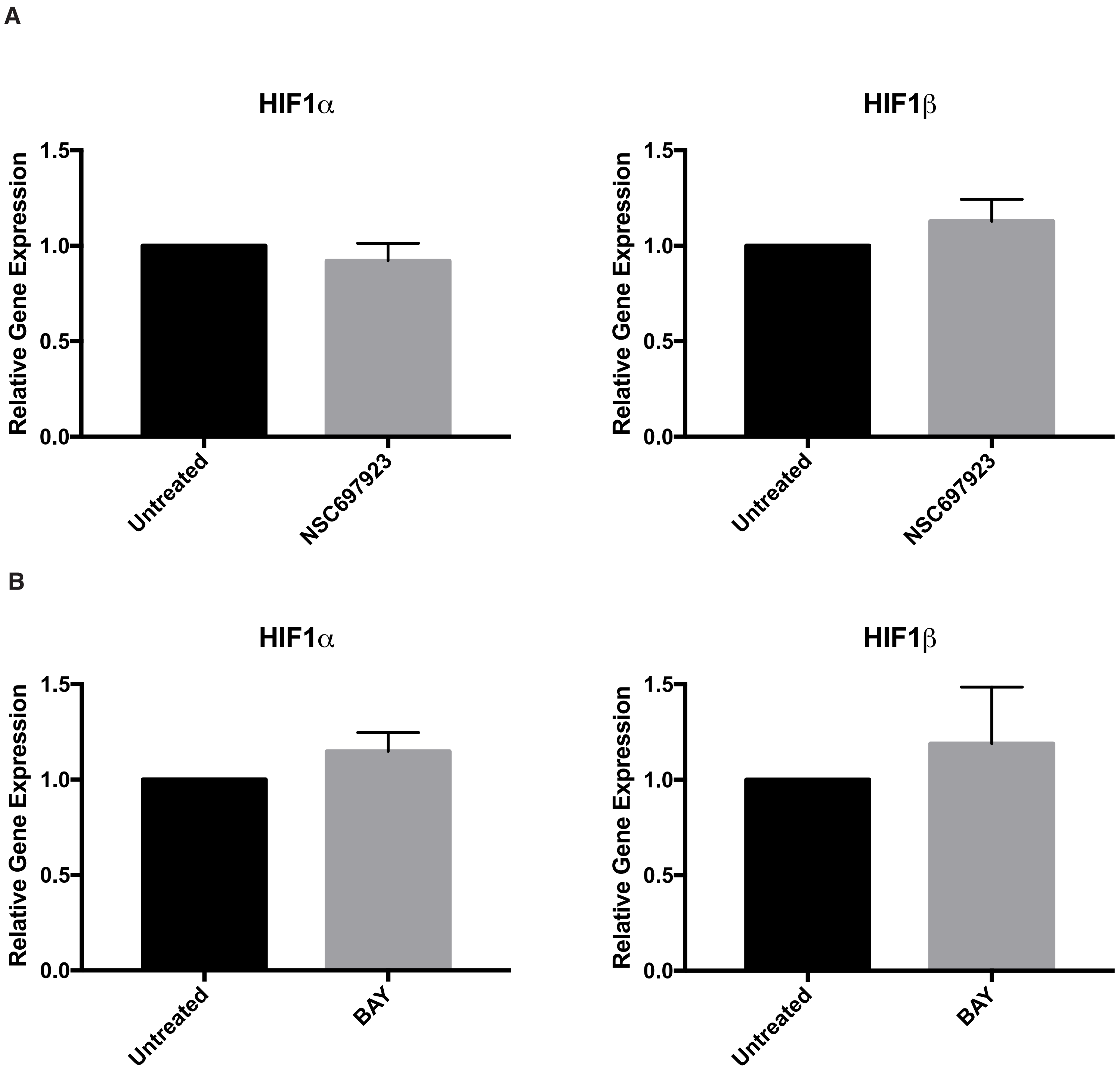

Supplementary Figure S4

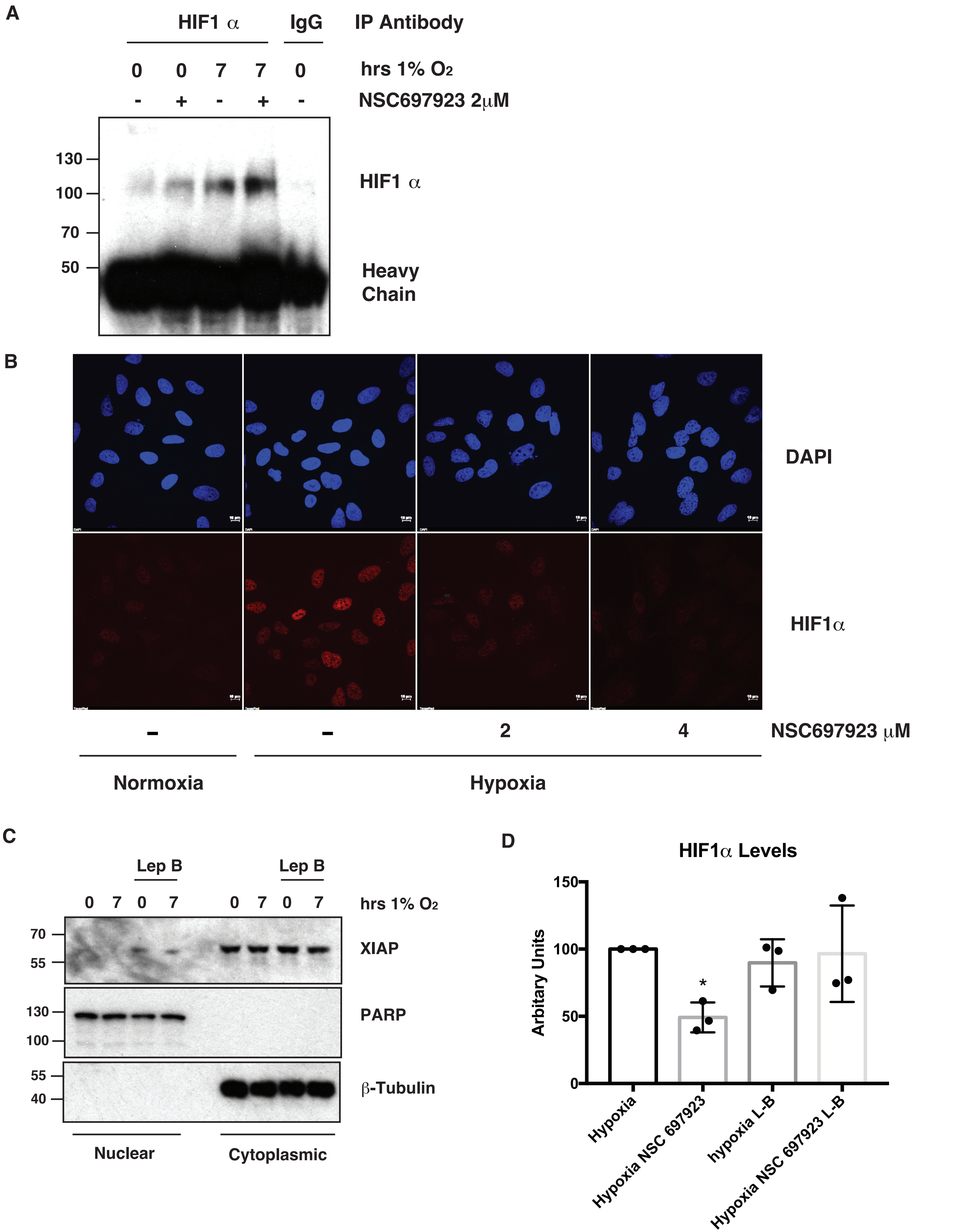

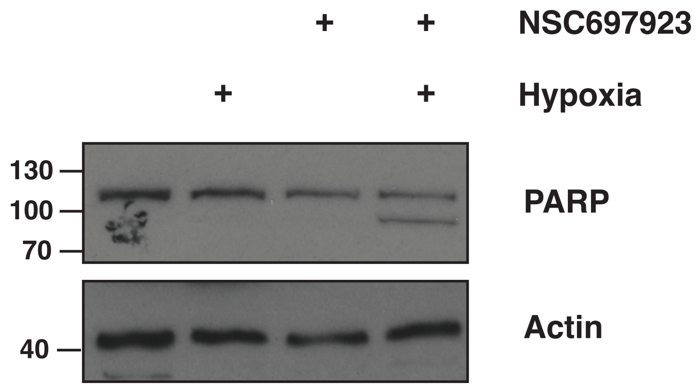
+
+
NSC697923
+
+
Hypoxia
130
100
70
PARP
40
Actin

Figure S1 XIAP does not target known regulators of HIF-1α

(A) HEK293 cells were transiently transfected with plasmids encoding HA-PHD1, HA-PHD2, HA-PHD3 or HA-VHL with His-tagged ubiquitin, and cotransfected with Flag-XIAP as indicated. 48 hours post-transfection ubiquitinated complexes were stabilized by the addition of MG132 5h prior to harvesting. Ubiquitinated material was recovered from lysates by incubation with nickel-coated beads and analyzed by anti-HA western blotting (B) Biotinylated-HIF1α was expressed in HEK293 cells and cells were treated with the indicated concentrations of NSC697923 and BAY 11-7082 for 3 hours. HIF1α was recovered from lysates by incubation with streptavidin-coated beads and analyzed using antibodies directed against specific ubiquitin chains. (C) U2OS cells were treated with MG132 for 2 hours and then lysed in the presence or absence of UBQ-TUBEs as indicated. Complexes isolated using NiNTA beads and input samples (20%) were analyzed by immunoblotting with anti- HIF1α antibody. (D) U2OS cells incubated in 1% O_2 for 3 hours and then lysed in the presence or absence of TAB2-TUBEs or TAB2-AA-TUBEs as indicated. Complexes isolated using NiNTA beads and analyzed by immunoblotting with the indicated antibodies. (E) RCC4 were treated with 2uM NSC697923 as indicated before being lysed in the prescence of UBQ-TUBEs. Ubiquitin conjugates were isolated using NiNTA agarose beads and analysed by immunoblotting using a specific HIF1a antibody. NPD – NiNTA agarose pull-down.

Figure S2 HIF1 target gene expression is suppressed by Ubc13 Inhibition (A) Quantitative RT–PCR analysis of ANKRD37 and CAIX mRNA prepared from U2OS cells pre-treated with 15μM BAY 11-7082 for 30 min before being exposed to 1% O_2 for 7 hours. (B) Immunoblot analysis of U2OS cells transfected with siRNAs targeting Ubc13 or a non-targeting control. (C) Quantitative RT–PCR analysis of ANKRD37 and CAIX mRNA prepared from U2OS cells expressing siRNAs targeting Ubc13 or a control siRNA, and subsequently exposed to 1% O2 for 7 hours. (D) Quantitative RT–PCR analysis of ANKRD37 and CAIX mRNA prepared from HEK293 and PC3 cells pre-treated with 2μM NSC697923 or 15μM BAY 11-7082 for 30 min before being exposed

to 1% O_2 for 7 hours. All values are normalized to RPL13A mRNA and fold change calculated from control samples prepared in normoxic conditions. Statistical analysis using a one-way ANOVA using the Dunnett multiple comparison test, all samples are compared to the control hypoxic sample.

Figure S3 Ubc13 inhibition does not alter expression of HIF1α or HIF1β mRNA Quantitative RT–PCR analysis of HIF1α and HIF1β mRNA prepared from U2OS cells treated with 2μM NSC697923 or 15μM BAY 11-7082 for 7.5 hours. All values are normalized to RPL13A mRNA and fold change calculated from normoxic controls.

Figure S4 Suppression of Lys^{63}-linked polyubiquitination of HIF1α reduces HIF1α nuclear localization (A) HIF1α was precipitated from cytoplasmic extracts prepared from U2OS cells treated with 2μM NSC697923 and exposed to 1% O_2 as indicated. **(B)** U2OS cells pre-treated with 2μM or 4μM NSC697923 for 30min before being exposed to 1% O_2 for 3 hours. Cells were fixed and stained for immunofluorescence using the indicated antibodies. **(C)** Cytoplasmic and nuclear extracts were prepared from U2OS cells treated with 5 ng/ml Leptomycin B as indicated before being exposed to 1% O_2 for 3 h. **(D)** Quantification by densitometry of Figure 4D. Significance is calculated from the control hypoxic samples.

Figure S5 Ubc13 inhibition increases levels of cleaved PARP in hypoxic cells U2OS cells pretreated with 2μM NSC697923 for 30min before being exposed to 1% O_2 for 24 hours. Lysates were immunoblotted using the indicated antibodies.
